# Treatment of Dysentery by Large Doses of Ipecac

**Published:** 1876-06

**Authors:** Thos. D. Williams


### TREATMENT OF DYSENTERY BY LARGE DOSES OF IPECAC.

By THOS. D. WILLIAMS, M.D.

The cases I now report occurred between the 27th of August and the 3d of October, 1875, and were found in an area of country four miles long by three wide. Previous to and during their outbreak, the malarious troubles were very prevalent in the same district, but few families escaping, and, in numerous instances, nearly every member of the same family was attacked simultaneously, and with some few a diarrheal tendency was observed. In accordance with the rules given for the administration of ipecac, water and fluids of all kinds were denied the patient for two or three hours before and after taking the medicine.

CASE I.—Mrs. D. L., aged thirty-eight, attacked August 27th, with mild disenteric symptoms or discharges, tormina and tenesmus; had been complaining with diarrhea for about five days; loss of appetite and general languor; had some eight or ten discharges in two or three hours from the time she was taken till I first saw her, about nine o'clock A.M. At this time, the evacuations were frequent, bloody, and mucus; high fever, quick pulse, and general systemic distress. Gave sulph. morphia gr.ss and ipecac grs. xvi at one dose, followed by vomiting in one hour. Ordered morphia gr.ss to be taken in three hours. Bowels moved once. Repeated ipecac in same dose in six hours from first dose. Vomited in three hours; bowels moved soon after the vomiting; less blood and mucus. August 28th, I saw her at ten o'clock A.M. She had taken half a grain of morphia during the night; one evacuation during that time; no pain, blood or mucus. The fever lasted her about two days, due, doubtless, to malarial influences; it gave way under the proper treatment. There was no return of the pain or dysenteric discharges after the second vomiting. Dismissed.

CASE II.—Miss N. H., aged sixteen years, attacked September 14th, A.M., with severe dysenteric discharges, high fever, tenesmus, tormina and emesis. Saw her at eight A.M. Gave snlph. morphia gr. ¼ hypodemically, as there was great gastric irritability; repeated same dose in half an hour, as the discharges of blood and mucus were still frequent and painful; then gave ipecac grs. xii in pill, followed by vomiting in half an hour. Directed her to take half a grain of morphia if the bowels pained or moved frequently before I could visit her. Called at two P.M.; three actions since I left to this hour; had taken the morphia. Gave ipecac grs. xvi, followed by vomiting in three or four hours. From this time until I saw her on the 15th, nine A.M., there were two evacuations from the bowels; dysenteric discharges arrested; gave a few small doses of sulphate quinia; convalesence rapid; no return whatever of unpleasant symptoms. Case dismissed.

CASE III.—Miss M. R., stout, aged twenty-three years, attacked September 27th, with very violent dysenteric symptoms, frequent actions of blood and mucus, tormina, tenesmus, high fever, quick pulse, emesis, general systemic distress. Gave morphia gr. ss and ipecac grs. xx in one dose; vomited in about twenty minutes. Saw patient in three hours; up to this time had vomited again; bowels moved once; less pain, blood and mucus. Gave morphia gr. ss; directed her to take ipecac grs. xx in pill at three P.M.; this followed by vomiting in half an hour; the bowels did not move but twice from ten A.M. until three P.M., after second dose of ipecac. They did not move but three times from three P.M. until ten A.M. on the 28th, at which time I gave her morphia gr. ss, which completed the cure. Dysenteric discharges were promptly arrested after the second vomiting. Case dismissed.

CASE IV.—W. R. K., male, rather stout, aged nineteen years, attacked on the 28th, A.M. Saw him at three P.M. Had had some fifteen bloody and mucus discharges from early morning till this hour; tormina, tenesmus, high fever, quick pulse very marked; gave, at once, tincture opii, gtt. xxv; and ipecac grs. xx, in pill; vomited in one hour; bowels moved again; less pain; blood and mucus still present; gave half a grain of morphia. Saw him about night; the actions were not so frequent, but rather painful. Repeated the ipecac in same dose ; was followed by vomiting in three hours. Gave morphia gr.ss at ten P.M. Saw him at nine A.M. on the 29th. During the night there had been two evacuations, the last containing no blood or mucus, with no pain. There being some irritability of the stomach and bowels, I ordered sulph. morphia gr.ss, which completed the cure. There was no return of the pain or discharges. Did not see him again.

There was free perspiration in three of the above cases. I did not use as large doses of ipecac in the treatment of these cases as some physicians have done, but vomiting occurred in from twenty minutes to three or four hours in all the cases, which does not accord with the exoerience of other physicians, who gave it in large doses, and state that it seldom vomited.

The dysenteric symptoms were promptly arrested, followed by free, bilious, feculent actions, without pain, attended with general relaxation, and sense of relief of the entire system, without a return of the distressing symptoms.—*Louisville Medical News.*

